# Prevalence of cognitive impairment in HIV patients: vertical and horizontal transmission

**DOI:** 10.1590/1980-5764-DN-2021-0023

**Published:** 2022

**Authors:** Maria Rita Polo Gascón, Cauê Peter da Cruz Terra, Hestela de Lima Guerra, Carolina Fernandes Gualqui, Mara Cristina Souza De Lucia, Glaucia Rosana Guerra Benute, Luiz Augusto Marcondes Fonseca, Jorge Casseb, Jose Ernesto Vidal, Augusto César Penalva de Oliveira

**Affiliations:** 1 Universidade de São Judas Tadeu, São Paulo SP, Brazil.; 2 Universidade de São Paulo, Hospital das Clínicas, Departamento de Dermatologia, São Paulo SP, Brazil.; 3 Universidade de São Paulo, Hospital das Clínicas, Departamento de Psicologia, São Paulo SP, Brazil.; 4 Universidade de São Paulo, Faculdade de Medicina, Instituto de Medicina Tropical, São Paulo SP, Brazil.; 5 Instituto de Infectologia Emílio Ribas, Departamento de Neurologia, São Paulo SP, Brazil.

**Keywords:** AIDS Dementia Complex, HIV, Cognitive Dysfunction, Complexo AIDS Demência, HIV, Disfunção Cognitiva

## Abstract

Antiretroviral treatment has significantly increased the survival of patients infected with HIV-1. However, with increased survival, cognitive changes associated with HIV are frequently observed in this population. The clinical manifestations of HIV changes can vary as a result of several aspects, including the virus transmission route. Several studies have pointed out premature neurological changes in vertically infected patients, while the manifestation of cognitive damage in adults may take a longer time.

**Objective:**

The aim of this study was to verify the prevalence of cognitive changes in patients with HIV via vertical transmission after the highly active antiretroviral therapy and the cognitive performance of these patients compared to a group of sexually infected patients.

**Methods:**

A total of 48 patients were evaluated, 25 with vertical transmission and 23 with sexual transmission, between May 2013 and February 2015 at the Institute of infectology Emilio Ribas. Neuropsychological tests were applied to assess cognitive performance, scales to assess symptoms of anxiety and depression, and sociodemographic questionnaire.

**Results:**

The results demonstrate that the frequency of cognitive impairment in vertically transmitted patients was higher than in sexually transmitted patients.

**Conclusions:**

These findings suggest that the deleterious effects of the HIV virus on the development of the central nervous system reverberate more strongly than in patients who acquire it after adulthood.

## RESUMO.

O tratamento antirretroviral tem aumentado significativamente a sobrevida de pacientes contaminados pelo HIV-1. Entretanto, com o aumento da sobrevida, observam-se frequentemente alterações cognitivas associadas ao HIV nessa população. As manifestações clínicas das alterações do HIV podem variar em decorrência de diversos aspectos, entre eles a via de transmissão do vírus. Diversos estudos têm apontado alterações neurológicas prematuras em pacientes contaminados por via vertical, enquanto a manifestação de danos cognitivos em adultos pode levar um tempo maior.

**Objetivo:**

O objetivo deste estudo foi verificar a prevalência das alterações cognitivas em pacientes com HIV via transmissão vertical após a era da terapia antirretroviral altamente ativa e o desempenho cognitivo desses pacientes comparado ao de um grupo de pacientes contaminados por via sexual.

**Métodos:**

Foram avaliados 48 pacientes, sendo 25 com transmissão vertical e 23 com transmissão sexual no período entre maio de 2013 e fevereiro de 2015, no Instituto de Infectologia Emílio Ribas. Foram aplicados testes neuropsicológicos para avaliar o desempenho cognitivo, escalas para avaliar sintomas de ansiedade e depressão e questionário sociodemográfico.

**Resultados:**

Os resultados demonstraram que a frequência de comprometimento cognitivo em pacientes contaminados via transmissão vertical foi maior do que naqueles contaminados via transmissão sexual.

**Conclusões:**

Essas descobertas sugerem que os efeitos deletérios do vírus HIV na formação do sistema nervoso central repercutem de forma mais acentuada do que em pacientes que o adquiriram após a vida adulta.

## INTRODUCTION

HIV-1 is a viral infection that belongs to the retrovirus family. This virus family has an enzyme called reverse transcriptase, in which, once inside the host cell, it participates in the process of converting viral RNA into DNA. After the conversion of its genetic material, it is incorporated into the cell’s DNA nucleus by the integrase enzyme^1^. Despite having no direct infection in neurons, HIV-1 infection has been associated with neuronal loss in several regions of the brain such as the frontal cortex, periventricular regions, and nuclei of the base, among others^2,3^. Even though neuronal losses are not directly associated with the infection of neurons, findings indicate that HIV-1 infects the central nervous system (CNS) support cells, such as microglia and astrocytes. This cellular group is extremely important for maintaining the interstitial environment of nervous tissue, since it coordinates immune responses and synthesis and release of amino acids used by neurons, controls the balance of the ions responsible for the action potential in the neuronal membrane, and points out infection of these cells that is thought to lead to inflammation of the nervous tissue^4^.

HIV-associated neurocognitive disorder (HAND) is frequent, with an estimated incidence of 15–50% among patients^5^. Neurological manifestations are extremely polymorphic and can compromise both CNS and peripheral nervous system (PNS), in addition to favoring the emergence of secondary diseases caused by pathogens (infectious or neoplastic), due to immunosuppression. High rates of severe and progressive encephalopathy were commonly seen at the beginning of the epidemic (50–90%), however, improving antiretroviral treatment reduced viral load, decreasing the number of infected cells in the CNS, and slowing the progress of neurological disorders caused by HIV^6^.

Sexual transmission is also referred to as horizontal transmission, as it occurs through direct contact with the infected agent; thus, in addition to the sexual route, it can be propagated by transfusion of contaminated blood and the use of unsterile cutting or piercing instruments. Vertical transmission, on the other hand, is the process in which the virus passes from the mother to the baby, which may occur during pregnancy, labor, or through breastfeeding^7^. HIV infection in adults is an infection that affects the brain fully formed and mature, while in children, the infection manifests itself as a progressive encephalopathy^8^. Neurological disorders resulting from the HIV virus in children who have been infected via vertical transmission vary from 50 to 90%, depending on the site of involvement and the period of infection. The damages are extremely polymorphic and can affect practically all sectors of the CNS, as well as in the infection acquired by sexual contact^6^.

Since it is observed that HIV-1 indirectly infects neurons and produces biochemical changes that lead to inflammation of the nervous tissue, the clinical manifestation of the changes caused by HIV is extremely polymorphic and depends, among other factors, on the through contagion; this study aimed to verify the prevalence of cognitive changes in patients with HIV via vertical transmission after highly active antiretroviral therapy (HARRT) and the cognitive performance of these patients compared with a group of HIV patients infected by sex, since there are no studies in the literature that directly compare these two groups.

## METHODS

The sample of this study is part of the cross-sectional research entitled “Prevalence and factors associated with cognitive changes in HIV patients” (approval number 14227913.0.0000.0061). The project was carried out in patients infected with the HIV-1 virus, in a segment at the Institute of infectology Emilio Ribas (IIER) from May 2013 to February 2015. IIER is a tertiary educational institution and a referral center for patients with infectious diseases in Brazil. Through a database of 575 patients, 48 were selected, 25 with vertical transmission and 23 with sexual transmission. Inclusion criteria were considered to be patients aged 18 years or above, with a minimum education of 4 years, and agreement to participate in the project by signing the free and informed consent form (ICF). Patients with concomitant diagnosis of active opportunistic neurological diseases (e.g., cerebral toxoplasmosis, neurotuberculosis, cryptococcal meningitis, and progressive multifocal leukoencephalopathy), previously documented conditions (i.e., traumatic, metabolic, vascular, or degenerative) that hinder the assessment of neurological symptoms and signs (disease) (e.g., Alzheimer’s disease or vascular dementia, and diabetic neuropathy), the use of psychoactive substances, and inability to understand the contents necessary for neurological assessment were excluded.

After applying the inclusion and exclusion criteria, the remaining patients were divided according to the form of transmission (previously identified through medical history). All HIV-positive patients infected via vertical transmission were selected, then the HIV-positive group via sexual transmission was established using the mean age defined by analysis of covariance (ANCOVA).

The demographic and epidemiological data evaluated were sex, age, date of diagnosis of HIV infection, and mechanism of infection transmission; clinical data; current and historical use of antiretrovirals; laboratory data; and CD4+ lymphocyte count, quantification of HIV-1 viral load, hemoglobin, glucose, lipid profile, and the presence of hepatitis B or C virus infection. The laboratory results closest to clinical evaluation (up to 3 months before) were considered.

Questionnaires were also applied to rule out possible neurocognitive changes secondary to depression (Beck Depression Inventory [BDI]^9^ and Hospital Anxiety and Depression [HAD] Scale)^10^. The International HIV Dementia Scale (IHDS)^11^ was applied, which assessed patients’ motor agility, psychomotor speed, and memory.

The neuropsychological profile of the patients was determined by a formal neuropsychological assessment comprising the following instruments: Intellectual Functions: Vocabulary and Matrix Reasoning subtest using the WAIS-III scale^12^; Attention/Speed of Information Processing: Color Trail Tests 1 and 2^13^; Trail Making Tests A and B^14^, and subtest Codes of the WAIS-III scale^12^; Memory: Short Term or Operational: subtest Direct and indirect digits of the WAIS-III scale^12^; Immediate and Late Visuals/Rey Complex Figures^15^; Executive Function/Categorical Verbal Fluency: FAS and Animals^16^; and Motor skill: Grooved Pegboard and Finger Tapping Test^17^. For each neuropsychological test, the Z score was calculated using age and/or education, adjusted with the normative data for each test.

The results of the neuropsychological assessment were classified according to Frascati’s criteria^18^: asymptomatic neurocognitive impairment (ANI — includes cognitive impairment involving at least two cognitive domains, performance of at least 1 standard deviation [SD] below the mean for norms on neuropsychological tests, and the cognitive impairment does not interfere with everyday functioning); mild neurocognitive disorder (MND — includes cognitive impairment involving at least two cognitive domains, performance of at least 1 SD below the mean for norms on neuropsychological tests, and the cognitive impairment produces at least mild inference in daily functioning); and HIV-associated dementia (HAD — includes marked cognitive impairment involving at least two cognitive domains, performance of at least 2 SD below the mean for norms on neuropsychological tests, and the cognitive impairment produces marked interference with day to day functioning).

To compare neuropsychological performance between groups, analyses of variance were performed for independent samples with one factor (one-way ANOVA) and linear regression analyses to identify possible covariates associated with the participants neuropsychological performance (gender, age, education, and depression). After the establishment of the covariables, an analysis of three groups (ANCOVA) was carried out in order to eliminate the effect due to these covariables, therefore reducing the error variance. The effect size of the comparisons was estimated by Cohen’s d. The magnitude of the effect was interpreted as small when the value was up to 0.2, moderate when the value was at 0.5, and large when ≥0.8. The results of Cohen’s d analysis highlighted the clinical significance of the results, not just focusing on statistical significance.

## RESULTS

Table 1 shows the sociodemographic and clinical data of the two groups. Statistical differences were observed in relation to sex, age, and education. Thus, it was identified that patients with vertical transmission had shorter schooling, with an average of 10.52 years. In addition, there is a higher frequency of female patients (56%). On the other hand, the average length of schooling is higher (12.7 years) in the sexually transmitted group, and the frequency of female patients is significantly lower (17.4%). Regarding CD4, viral load, and the presence of symptoms of depression and anxiety, no statistical differences were observed.

**Table 1:** Sociodemographic and clinical data.

		Transmission form: vertical (n=25)	Transmission form: sexual (n=23)	F	p-value
Gender, n (%)					
	Male	11 (44)	19 (82.6)	8.769	0.005
	Female	14 (56)	4 (17.4)		
Age (years)		20.20 (2.16)	23.57 (2.40)	26.03	<0.001
Education (years)		10.52 (1.78)	12.17 (2.53)	6.92	0.012
CD4		535 (313)	574 (246)	0.220	0.641
Viral load undetectable (<copies 50 mm), n (%)		10 (40)	13 (59)	0.252	0.618
Duration HIV infection (years)		20.47 (2.24)	5.36 (2.32)		0.027
BDI-II		13.28 (10.97)	16.43 (9.78)	1.097	0.300
HAD – anxiety		6.40 (4.20)	7.69 (4.12)	1.158	0.288
HAD – depression		5.44 (3.17)	5.43 (3.24)	0.945	0.336

CD4: cluster of differentiation 4; BDI: Beck Depression Inventory; HAD: Hospital Anxiety and Depression Scale.

In Table 2, the absence of cognitive alterations was identified in 34.7% of the patients infected via sexual transmission, while in the vertical transmission group, only 12% did not show any decline. Regarding asymptomatic cognitive alterations, patients in the vertical transmission group presented 48% while those in the sexually transmitted group presented 60.8%. It was also possible to observe a prevalence of 40% of mild/moderate cognitive alterations in patients with vertical transmission and a prevalence of only 4.3% in patients with sexual transmission. When comparing the distribution of ANI and MDN alterations within the sexually transmitted group, a discrepant difference can be observed in relation to frequency, while only 1 patient presented the symptomatic form, 14 presented the asymptomatic form. However, when comparing the frequency of ANI in the vertical transmission group (12) with the frequency of MDN (10), it is observed that the values are homogeneous.

**Table 2:** Frequency of cognitive changes.

	Transmission form: vertical	Transmission form: sexual	F	p-value
No change, n (%)	3 (12)	8 (34.7)	9.244	0.004
ANI, n (%)	12 (48)	14 (60.8)		
MND, n (%)	10 (40)	1 (4.3)		

ANI: asymptomatic neurocognitive impairment; MND: mild neurocognitive disorder.

From the data shown in Table 3, it is possible to verify that the test that showed a significant difference between the two groups was that of the complex figure of Rey and, when compared, the vertical group performed less than the sexual group. The Rey complex figure test is applied in three stages: copy of the figure, immediate evocation, late evocation. The p-values were significant only in the immediate recall (p<0.025) and in the late recall (p<0.026). These results indicate that the most significant difference in performance between the two groups is restricted to immediate and late visuospatial memory and nonverbal memory. Despite not showing statistical significance, some other tests showed small magnitude values, such as RAVLT (recognition), Finger Dominant Hand, Estimated Intellectual Function, Trail Making A, Color Trail Test 2, and FAS.

**Table 3:** Performance in the battery of neuropsychological tests.

Neurocognitive domains		Transmission form: vertical (N=25)	Transmission form: sexual (N=23)	F	p-value	D
Memory	RAVLT (immediate)	28.78 (23.59)	32.17 (23.56)	0.287	0.595	0.16
	RAVLT (post-interference)	33.78 (28.55)	31.53 (26.56)	0.268	0.608	0.16
	RAVLT (late)	31.39 (26.62)	31.75 (20.26)	0.082	0.776	0.09
	RAVLT (recognition)	39.72 (32.94)	28.62 (28.80)	1.590	0.214	0.38
	Rey (copy)	23.63 (26.87)	45.43 (24.99)	0.843	0.364	0.18
	Rey (immediate)	31.12 (31.43)	60.34 (31.09)	5.409	0.025	0.28
Visuoconstruction	Rey (late)	24.56 (29.51)	53.43 (28.11)	5.34	0.026	0.71
Motor skills’	Finger dominant hand	23.30 (29.86)	13.95 (22.36)	3.47	0.069	0.35
	Finger nondominant hand	15.72 (22.74)	19.89 (26.86)	0.039	0.843	0.06
	Grooved dominant hand	42.20 (30.23)	50.78 (30.39)	0.008	0.931	0.03
	Grooved nondominant hand	49.88 (29.59)	48.47 (18.97)	0.00	0.994	0.00
Processing speed	Codes	53.08 (25.26)	57.91 (23.34)	0.133	0.718	0.11
Intellectual function	QI estimated	36.92 (22.51)	50.00 (25.32)	1.50	0.226	0.37
	Vocabulary	41.56 (27.46)	55.00 (31.71)	0.106	0.746	0.10
	Matrix reasoning	59.00 (25.12)	66.00 (22.55)	0.106	0.747	0.10
Attention and information processing	Trail Making A	29.20 (21.94)	31.44 (24.69)	0.531	0.470	0.22
	Trail Making B	17.64 (21.14)	33.52 (32.42)	0.195	0.661	0.13
	TTC 1	53.15 (23.09)	65.00 (23.30)	0.278	0.601	0.16
	TTC 2	47.72 (21.99)	57.91 (24.26)	0.462	0.500	0.21
Executive function	FAS	20.76 (18.22)	23.31 (19.09)	0.705	0.406	0.26
	Animals	57.87 (54.71)	60.00 (31.48)	0.054	0.817	0.07
Cognitive screening	IDHS	11.14 (1.08)	11.37 (0.77)	0.043	0.837	0.06

RAVLT: Rey Auditory-Verbal Learning Test; TTC: Color Trail Test; IDHS: International HIV Dementia Scale.

## DISCUSSION

The main findings of this study point to (i) a higher prevalence of cognitive changes in patients with vertical transmission (88%); (ii) significant difference in the Rey Complex Figure test, with values of p<0.025 for immediate recall and p<0.026 for late recall; and (iii) significant difference in sex, age, and education between the two groups.

In this study, 88% (48% ANI form and 40% MND form) of patients in the vertical transmission group had cognitive impairments, compared to 65.1% (60.8% ANI form and 4.3% MND form) in the sexual transmission group. In addition to the higher prevalence of individuals with cognitive changes in the vertical transmission group, these results indicate a particular trend: there is an inversion in the magnitude of the frequency values of the groups in each category of diagnosis, showing that the vertical transmission group has a higher cognitive decline, as it has the lowest prevalence of individuals without cognitive changes and the highest with changes in the MND form. In the sexually transmitted group, the opposite is observed.

Regarding the distribution of ANI with MND within each group, a particular difference is observed. The sexually transmitted group has a frequency of only 4.3% in the MDN and a frequency of 60.8% in the ANI, resulting in outliers between the two categories. However, when comparing the ANI frequency of the vertical transmission group (48.3%) with the frequency of MND (40%), it is noted that the values are equivalent. These frequency differences between ANI and MND analyzed show that patients with vertical transmission evolve more frequently from asymptomatic cognitive changes to mild/moderate symptomatic cognitive changes.

A longitudinal study that compared the evolution of HAND in seropositive patients through neuropsychological tests identified that subjects who already had ANI had a higher risk (two to six times) of developing MND changes in a shorter period, when compared to subjects who did not have any type of cognitive impairment, indicating that the first cognitive impairments, even if asymptomatic and, therefore, not noticed by the patient, can predict a faster symptomatic evolution of the HAND^19^. Although ANI is a predictor of the fastest evolution to MND, the simple clinical diagnosis of ANI should not be taken as the only predisposing clinical factor for the evolution to MND. Factors such as age, sex, and schooling are also associated with rapidity toward cognitive decline.

Most neurological studies have focused on the adult population, whose acquisition of infection was mostly through sexual transmission; however, significant differences were observed between the CNS of individuals who contracted the virus in childhood via vertically and adults who contracted it sexually^20^. The damage caused by HIV in the CNS of children is characterized by a failure to reach the markers of child neurological development, or by the loss of already acquired functions, leading in some cases to acquired microcephaly, nervous tissue atrophy, and motor deficits in the pyramidal tract^20,21^. Neuroimaging studies indicate that the most common neuropathological alterations in HIV-positive children with cognitive alterations include generalized cortical and subcortical atrophy, which are observed through the enlargement of the ventricles and/or expansion of the sulci between the cerebral turns, in addition to calcification of the basal ganglia and the periventricular frontal white matter^21^.

One study found that HIV-seropositive children, when compared to a control group, had lower performance in all cognitive domains, but especially in working memory, attention, and processing speed^21^. Another study found that even in asymptomatic HIV-positive children, when compared to a control group, they also had lesser performance in neuropsychological tests, with this sample found declines in visuospatial ability, visuospatial memory, semantic memory, and IQ^22^. A meta-analysis pointed out that vertical transmission patients show greater cognitive decline in working memory, visuospatial memory, processing speed, and executive function^23^.

In our study, the test that demonstrated a significant difference between the two groups was the test of the complex figure of Rey, who assesses visuospatial memory. This result indicates that the most significant difference in performance between the two groups is restricted to immediate and late visuospatial memory and nonverbal memory. Thus, based on previous studies^24,25^, which pointed out the deterioration of the cells of the hippocampus due to the toxicity of HIV products^26^ and, as a consequence, the decline of some memory functions that depend on the hippocampus, such as visuospatial memory, which was the most affected domain in the vertical transmission group in this study, it is possible to speculate that the transmission group vertical, who has a longer time of HIV infection, has a higher level of hippocampal contamination. In addition, it has already been pointed out that the hippocampus may be the most sensitive area for HIV infection in the CNS and be one of the most responsible for HAD, since it has been observed that neurons of hippocampal formation in patients who developed AIDS were stunted^24^.

HIV-positive children and adolescents are at increased risk of both CNS sequelae and mental disorders owing to a number of factors, including the impact of HIV infection on the brain, social determinants of health (e.g., poverty and orphanhood), and psychosocial stressors related to living with HIV^27^. The majority of children with HIV-associated neurological disease are infected by maternal–fetal transmission. Through this route, there is an increased risk of irreversible brain damage, including cerebral atrophy, intracerebral calcifications, and microcephaly, as well as various degrees of developmental delay and cognitive impairment^28^. ­Clinical features include loss or failure to achieve appropriate developmental milestones, impaired brain growth, and global or selective impairments in cognitive, language, motor, attention, behavior, and social skills that may affect day-to-day functioning^28^. ­Antiretroviral therapy (ART) does not eliminate HIV-associated CNS manifestations. ART works mainly to prevent HIV replication and reduce the progression of cell damage; by delaying the use of ART, profound irreversible inflammatory changes may occur, contributing to the emergence of severe neurocognitive deficits^28^.

The results demonstrate that the frequency of cognitive impairment in patients via vertical transmission was higher than in patients via sexual transmission. Although both groups are matched for age, the time of infection in the vertical group is much longer when compared, presenting a statistically significant (p<0.027). These findings suggest that the deleterious effects of the HIV virus on the developing CNS reverberate more strongly than in patients who acquire it after adulthood. In this research, it was not possible to conclude whether the cognitive changes observed in the vertical group are due exclusively to the presence of the virus or whether it can also be attributed to some neurodevelopmental disorder; however, given the low survival of these patients, it is possible to assume that the virus influences brain maturation. Thus, the seriousness of public politics for the prevention of HIV infection is highlighted, mainly through vertical transmission, emphasizing the importance of neuropsychological monitoring of already infected patients, with a focus on improving the quality of life.

As limitations, we have the small number of patients, requiring more studies with greater sampling power for future generalizations. Formal IQ data related to school performance and premorbid level are also important areas of analysis that were not explored in this study and would be useful to provide an overview of developmental milestones for patients infected via vertical transmission. Longitudinal data on the cognitive performance of both groups are needed so that inferences about the progression of HAND can be made.
